# Psychological and Physiological Stress and the Onset and Course of Graves’ Disease: A Systematic Review

**DOI:** 10.7759/cureus.113609

**Published:** 2026-07-29

**Authors:** Sheimaa Nasreldein Nureldaim Ahmed, Ali Hadi M Alhajri, Eltayeb Osman Elfaki Omer, Hadia Abdalla Elneel Rabah, Samih Abdelmutalab Mohamed Abdalla, Rihab Abdelhafiz Hassan khattab, Eikhlas Nasreldin Osman Ali, Eman Barakat Bayonis

**Affiliations:** 1 General Medicine, Sheikh Khalifa General Hospital, Ummalquwain, ARE; 2 Endocrinology, Najran Armed Forces Hospital, Ministry of Defense Health Services, Najran, SAU; 3 Endocrinology, Dr. Sulaiman Al Habib Hospital, Riyadh, SAU; 4 Medical Services, Ministry of Interior, Arar, SAU; 5 Internal Medicine, Al Hammadi Hospital, Riyadh, SAU; 6 Pathology, Khartoum University, Khartoum, SDN; 7 Medicine and Surgery, Elrazi University, Khartoum, SDN; 8 Medicine and Infectious Diseases, Jazan Specialist Hospital, Jazan, SAU

**Keywords:** autoimmune thyroid disease, graves’ disease, hyperthyroidism, hypothalamic-pituitary-adrenal axis, psychological stress, stressful life events, systematic review

## Abstract

Graves’ disease (GD) is the most common cause of autoimmune hyperthyroidism, and stress has long been implicated in its onset. Whether stress genuinely triggers thyroid autoimmunity or is an epiphenomenon of evolving hyperthyroidism remains unclear. This systematic review appraised original observational studies examining the association between psychological and physiological stress and the onset and clinical course of GD.

Following the Preferred Reporting Items for Systematic Reviews and Meta-Analyses (PRISMA) 2020 statement, MEDLINE/PubMed, Embase, Scopus, Web of Science, PsycINFO and the Cochrane Library were searched from database inception to June 19, 2026. Eligible studies were original case-control or cohort investigations assessing stress exposure with validated instruments in patients with confirmed GD relative to a control or comparison group. Reviews, case reports, conference abstracts, and preprints were excluded. Methodological quality was appraised with the Newcastle-Ottawa Scale (NOS), a validated nine-point instrument for non-randomized studies rating selection, comparability, and exposure or outcome ascertainment, with predefined thresholds used to classify risk of bias. Certainty of evidence was rated using Grading of Recommendations Assessment, Development and Evaluation (GRADE). Substantial heterogeneity in exposure instruments, comparators, and outcome metrics precluded meta-analysis, so the evidence was synthesized narratively.

Thirteen studies, enrolling at least 2,867 participants and including 1,134 patients with confirmed GD, met the eligibility criteria. Most retrospective case-control studies reported a significantly higher burden of negative life events in the 12 months preceding GD diagnosis than in controls, with large effect estimates and a female-predominant association. Where patients with non-autoimmune toxic nodular goitre served as comparators, the excess was confined to the autoimmune group. Three studies linked stress to disease recurrence and to a poorer response to antithyroid drug therapy, again chiefly in women. Conversely, the prospective cohort and a chronic endogenous stress study found no association with de novo thyroid autoimmunity or incident GD. Overall certainty of evidence was very low, principally because of the predominantly retrospective designs with attendant recall bias and reverse causation, inconsistency between retrospective and prospective findings, and imprecision from small samples.

Retrospective evidence supports an association between antecedent stress and GD onset and, to a lesser extent, its course in genetically predisposed individuals, particularly women, while the limited prospective evidence is inconsistent with this association. Adequately powered prospective cohort studies employing standardised stress instruments and objective stress biomarkers are needed before a causal role can be affirmed.

## Introduction and background

Graves’ disease (GD) is an organ-specific autoimmune disorder and the leading cause of hyperthyroidism, with an estimated incidence of 20-50 cases per 100,000 persons per year and a marked female preponderance, most cases presenting between the third and sixth decades of life [1]. The hallmark of the disease is the production of stimulating autoantibodies, which are immune proteins that mistakenly target the body’s own tissues and are directed against the thyrotropin (thyroid-stimulating hormone (TSH)) receptor. These antibodies mimic the action of TSH and drive unregulated synthesis and release of thyroid hormone, producing diffuse goiter and, in a substantial minority, orbitopathy [2]. Susceptibility is polygenic and is shaped by a well-characterized set of environmental and lifestyle modifiers, including cigarette smoking, iodine intake, selenium status, infection and pregnancy, which are thought to act upon a permissive genetic background to precipitate a loss of immune tolerance, the normal state in which the immune system refrains from attacking self-tissue [3]. Among these putative modifiers, psychological and physiological stress has attracted enduring interest, in part because patients themselves frequently attribute the emergence of their illness to an antecedent period of adversity.

The biological plausibility of a stress-autoimmunity link rests on the cross-talk between the neuroendocrine and immune systems. Acute and chronic stressors activate the hypothalamic-pituitary-adrenal (HPA) axis and the sympathoadrenomedullary system, releasing glucocorticoids and catecholamines that reprogram immune function [4]. Repeated or sustained activation imposes an allostatic load, the cumulative physiological cost of adapting to persistent stress, whose mediators exert complex bidirectional effects on innate and adaptive immunity [5]. In thyroid autoimmunity specifically, stress hormones are proposed to shift the balance between the two principal arms of the helper T-cell response: the T-helper 1 (Th1) arm, which drives cell-mediated immunity, and the T-helper 2 (Th2) arm, which promotes antibody production. A shift favoring Th2 responses, together with impaired activity of regulatory T cells that normally restrain self-reactive lymphocytes, could favor the antibody-mediated process that characterizes GD; a rebound of immune activity following periods of stress-induced immunosuppression has likewise been invoked [6].

Clinical interest in this relationship long predates its formal study, with descriptions of goiter and thyrotoxicosis arising after fright, bereavement or protracted hardship recurring throughout the nineteenth- and twentieth-century literature. The modern evidence base is dominated by retrospective case-control studies quantifying stressful life events in the months preceding diagnosis, the most influential of which reported a several-fold excess of negative life events among newly diagnosed patients relative to controls [7]. Such designs are inherently vulnerable to recall bias and to reverse causation, since the prodrome of hyperthyroidism may itself generate or amplify the perception of stress. The single substantial prospective investigation to address the question found no association between stress exposure and the de novo development of thyroid autoimmunity [8]. The evidence is therefore genuinely conflicting, and no consensus exists as to whether stress is a causal trigger, a disease-modifying influence, or an artefact of study design.

This question has been examined previously. A systematic review and meta-analysis published in 2023 pooled nine case-control and four cohort studies and reported a large summary effect size, concluding that stress is an important factor in GD onset [9]. That synthesis nevertheless left several matters unresolved: it searched only to January 2023; it did not restrict eligibility to populations in which GD could be separated from other causes of thyrotoxicosis; it pooled heterogeneous exposure instruments and outcome metrics into a single standardized effect estimate despite marked methodological diversity; and it did not formally rate the certainty of the evidence. The present review was therefore undertaken with an updated search to June 2026, strictly GD-specific eligibility criteria, separate appraisal of onset and clinical course, a transparent narrative synthesis in place of statistically questionable pooling, and formal GRADE certainty rating. Establishing whether stress genuinely contributes to GD carries direct clinical consequences: if confirmed, stress reduction and psychosocial support would become legitimate adjuncts to antithyroid therapy and relapse prevention, whereas if refuted, clinicians could confidently reassure patients who blame themselves for their illness. Accordingly, the objective of this review was to identify, appraise, and synthesize the original observational evidence linking psychological and physiological stress to the onset and clinical course of GD, and to delineate the methodological limitations that continue to constrain causal inference.

## Review

Materials and methods

Protocol and Reporting

This systematic review was designed, conducted, and reported in accordance with the Preferred Reporting Items for Systematic Reviews and Meta-Analyses (PRISMA) 2020 statement [10]. A written protocol specifying the review question, eligibility criteria, search strategy, data-extraction plan, risk-of-bias approach, and synthesis method was finalized by the review team before the literature search commenced. The protocol was not prospectively registered with the International Prospective Register of Systematic Reviews (PROSPERO); this is acknowledged as a methodological limitation, and the authors will register future review protocols prospectively

Eligibility Criteria

Studies were selected against a predefined Population, Exposure, Comparison, Outcome, and Study-design (PICOS) framework, summarized in Table 1. Eligible reports were original observational studies, either case-control or cohort in design, that assessed a defined stress exposure in patients with a clinically and biochemically confirmed diagnosis of GD and that included a control or comparison group. Studies were excluded if they were reviews, meta-analyses, editorials, commentaries, case reports, or case series, conference abstracts, or preprints without peer review; if they were conducted in animals or in vitro; if they lacked any comparison group; or if they did not distinguish GD from other causes of thyrotoxicosis in a manner permitting GD-specific inference.

**Table 1:** Eligibility criteria structured according to the PICOS framework.

Parameter	Inclusion criteria	Exclusion criteria
Population (P)	Patients with a confirmed diagnosis of Graves’ disease (clinical and biochemical hyperthyroidism, supported where reported by TSH-receptor antibodies, radioiodine uptake or ultrasonography), of any age or sex.	Non-autoimmune thyrotoxicosis without a separable Graves’ disease subgroup; undifferentiated *hyperthyroidism*; Hashimoto’s thyroiditis or euthyroid populations only.
Exposure (E)	Psychological or physiological stress assessed with a validated stressful-life-event instrument (e.g., Paykel interview, Holmes-Rahe scale, Life Experiences Survey) or a clearly defined stressful condition preceding onset or during follow-up	Stress neither measured nor operationally defined; exposure limited to depression or anxiety symptoms without a stress-event measure
Comparison (C)	Healthy controls, non-autoimmune hyperthyroid controls (e.g., toxic nodular goiter), within-cohort non-cases, or (for course outcomes) patients who relapsed versus those who remitted	No comparison or control group
Outcome (O)	Onset/incidence of Graves’ disease and/or clinical course (recurrence, exacerbation, or response to antithyroid drug therapy)	Outcomes unrelated to Graves’ disease onset or course
Study design (S)	Original case-control or cohort studies (prospective or retrospective), peer-reviewed	Reviews, meta-analyses, editorials, commentaries, case reports/series, conference abstracts, preprints, animal, or in-vitro studies

Information Sources and Search Strategy

MEDLINE/PubMed, Embase, Scopus, Web of Science, PsycINFO, and the Cochrane Central Register of Controlled Trials were searched from database inception to 19 June 2026, without language restrictions. The search combined controlled vocabulary and free-text terms for the exposure and the condition, using a Boolean structure of the form: (“stress, psychological” OR “stressful life events” OR “life change events” OR “emotional stress”) AND (“Graves’ disease” OR “toxic diffuse goitre” OR “autoimmune hyperthyroidism” OR “thyrotoxicosis”). The complete database-specific search strings, with field tags and result counts, are reported in the Appendix. Reference lists of retrieved articles and relevant reviews were hand-searched for additional eligible studies.

Study Selection

**Figure 1:**
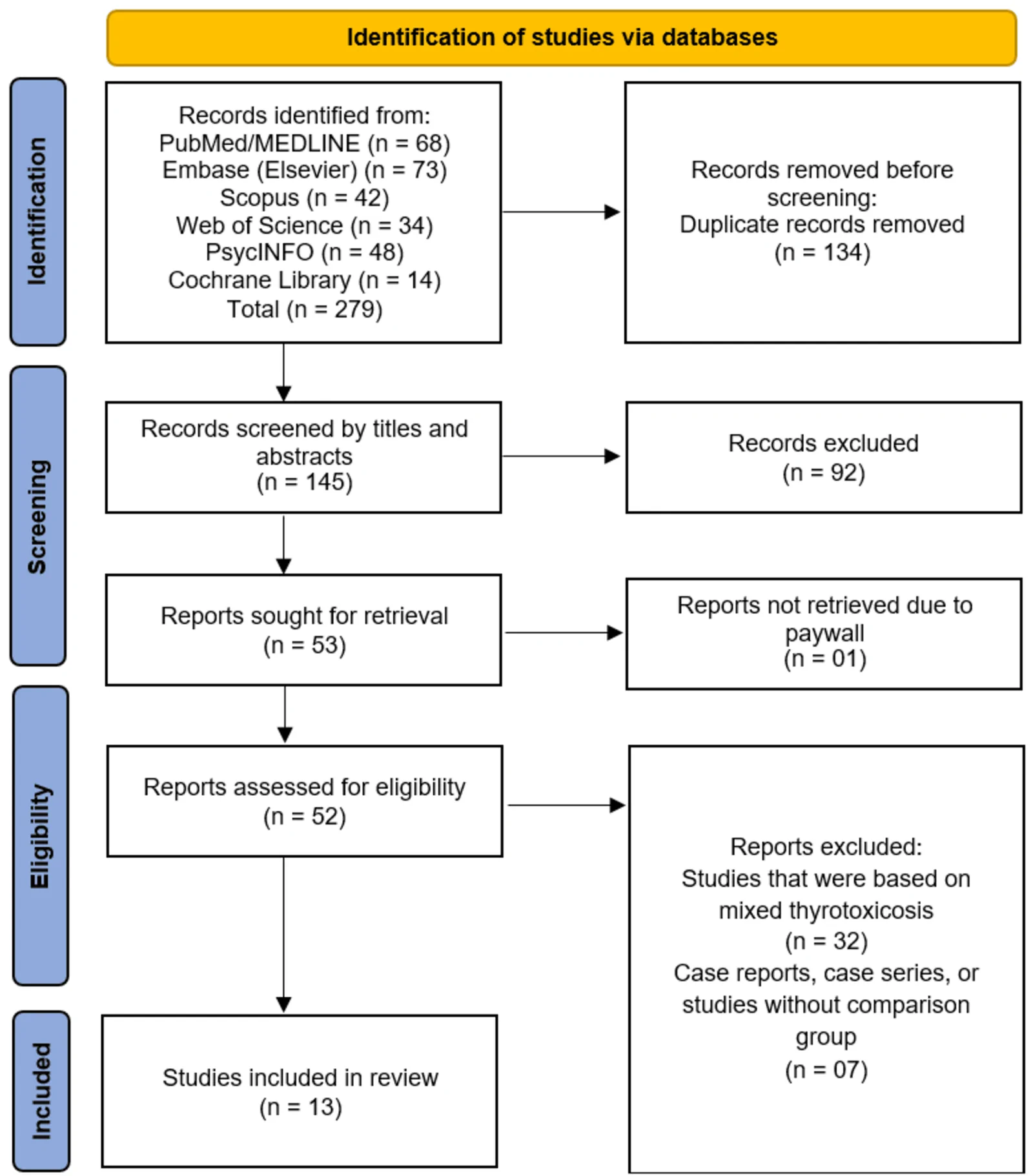
PRISMA 2020 flow diagram of study identification, screening, and inclusion. PRISMA, Preferred Reporting Items for Systematic Reviews and Meta-Analyses

Records from all sources were collated and de-duplicated. Two reviewers independently screened titles and abstracts and then assessed full texts against the eligibility criteria. Disagreements were resolved by discussion or, where consensus could not be reached, by adjudication from a third reviewer. Agreement between reviewers was high at both stages, although a formal chance-corrected agreement statistic was not calculated; this is acknowledged as a reporting limitation. The selection process is summarized in the PRISMA 2020 flow diagram (Figure 1).

Data Extraction

From each included study, two reviewers independently extracted the first author and year, country and setting, study design, number and characteristics of cases and comparators, the stress-assessment instrument and exposure window, the outcome assessed, the principal effect estimate reported with its precision where available, and the authors’ conclusions. Extracted data were compared, and discrepancies reconciled against the source article by consensus.

Risk-of-Bias Assessment

Methodological quality was appraised with the Newcastle-Ottawa Scale (NOS), which rates non-randomised studies across three domains: selection (maximum four stars), comparability (maximum two stars) and exposure or outcome ascertainment (maximum three stars), giving a maximum total of nine stars [11]. Design-appropriate versions of the scale were applied, with the exposure domain used for case-control studies and the outcome domain for cohort studies. Total scores were translated into risk-of-bias categories using thresholds specified in advance: seven to nine stars denoted low risk of bias, four to six stars moderate risk, and zero to three stars high risk. Each study was assessed independently by two reviewers, and disagreements were resolved by consensus.

Certainty of Evidence

The certainty of the body of evidence for each outcome was rated with the Grading of Recommendations Assessment, Development and Evaluation (GRADE) approach [12], in which bodies of observational evidence commence at low certainty and are rated down for risk of bias, inconsistency, indirectness, imprecision and publication bias, or rated up for a large magnitude of effect, a dose-response gradient, or the presence of plausible confounding that would reduce a demonstrated effect.

Data Synthesis

The included studies differed substantially in their stress-assessment instruments (semi-structured contextual interviews versus self-report inventories), exposure windows, comparators (healthy versus non-autoimmune hyperthyroid controls), and outcome metrics (event counts, mean impact scores, relative risks and categorical odds ratios). Because these effect measures are not commensurable and cannot be converted to a common scale without unverifiable assumptions, pooling them into a single summary estimate would have produced a statistically precise but clinically uninterpretable result; meta-analysis was therefore not performed. For the same reason, and because quantitative small-study effect diagnostics such as funnel-plot asymmetry and Egger’s test require a reasonably homogeneous set of comparable effect estimates and are unreliable with fewer than ten such studies per outcome, formal statistical assessment of publication bias was not undertaken; the potential for publication bias is instead addressed qualitatively in the GRADE appraisal and the limitations. The evidence was synthesized narratively following a structured approach: studies were first grouped by design (retrospective case-control versus prospective or cohort), since design determines vulnerability to recall bias and reverse causation; within each group, findings were tabulated by outcome domain (onset versus clinical course), the direction and statistical significance of each association were recorded, and results were compared across groups to establish where the evidence converged and where it diverged. Effect estimates are reported as presented in the primary studies, with their confidence intervals or P values where available.

Results

Study Selection

A total of 279 records were identified across PubMed/MEDLINE (*n* = 68), Embase (*n* = 73), Scopus (*n* = 42), Web of Science (*n* = 34), PsycINFO (*n* = 48), and the Cochrane Library (*n* = 14). After removal of 134 duplicates, 145 records were screened by title and abstract, of which 92 were excluded. Of the 53 reports sought for retrieval, one could not be accessed because of a paywall. Among the 52 reports assessed in full text, 39 were excluded: 32 because they reported mixed thyrotoxicosis without a separable GD subgroup, and seven because they were case reports, case series, or lacked a comparison group. Thirteen studies met the inclusion criteria (Figure 1). The relatively small final yield reflects the deliberately restrictive eligibility criteria rather than a limited search: the requirement for a diagnostically confirmed and separately analysable GD population, a stress exposure measured with a defined instrument, and a concurrent comparison group excluded the large body of literature on thyrotoxicosis generally, on psychiatric comorbidity in established GD, and on stress in autoimmune disease without thyroid-specific outcomes.

Study Characteristics

The included studies were conducted across nine countries (Sweden, Italy, Hong Kong, Serbia, Japan, Portugal, Taiwan, Turkey, and the Netherlands) and were published between 1991 and 2015. Eight used a case-control design, and five were cohort or prospective investigations. Collectively, they enrolled at least 2,867 participants, comprising 1,134 patients with confirmed GD across the 10 studies reporting case numbers, together with 856 explicitly enumerated comparison participants, 87 patients with panic disorder serving as a chronic endogenous stress cohort, and 790 euthyroid women at genetic risk followed prospectively. Several studies described matched control groups without stating the exact number of controls, so this cumulative figure represents a conservative minimum. Sample sizes of confirmed GD cases ranged from 31 to 230. Stress was operationalized through validated life-event instruments in every study, most commonly semi-structured interviews (Paykel; Holmes-Rahe) or self-report inventories (the Life Experiences Survey). Study characteristics are presented in Table 2, and the principal findings and direction of association in Table 3.

**Table 2:** Characteristics of the 13 included studies. GD, Graves’ disease; TNG, toxic nodular goiter; F, female; M, male; ATD, antithyroid drug; LES, Life Experiences Survey; PANAS, Positive and Negative Affect Schedule; AITD, autoimmune thyroid disease; pt-yr, patient-years

Study (year) [ref]	Country	Design	Participants (GD; comparison)	Stress instrument	Outcome
Winsa et al. (1991) [7]	Sweden	Population-based case-control	208 GD; 372 matched controls	Mailed life-event questionnaire	Onset
Effraimidis et al. (2012) [8]	Netherlands	Prospective nested case-control	790 euthyroid women (AITD relatives)	Life events, daily hassles, PANAS	De novo autoimmunity
Sonino et al. (1993) [13]	Italy	Case-control	70 GD; matched controls	Paykel interview	Onset
Kung (1995) [14]	Hong Kong	Case-control	95 GD; matched controls	Self-report life-event questionnaire	Onset
Radosavljević et al. (1996) [15]	Serbia	Case-control	100 GD; 100 matched controls	Paykel interview	Onset
Matos-Santos et al. (2001) [16]	Portugal	Case-control	31 GD; 31 TNG; 31 controls	Life-event interview	Onset
Topcu et al. (2012) [17]	Turkey	Case-control	45 GD; 24 TNG; 36 controls	Holmes-Rahe; LES	Onset
Lee et al. (2003) [18]	Taiwan	Case-control	GD; controls	Life-event questionnaire	Onset
Yoshiuchi et al. (1998) [19]	Japan	Case-control	228 GD (182 F, 46 M); controls	Self-rated questionnaire	Onset
Chiovato et al. (1998) [20]	Italy	Cohort (endogenous stress)	87 panic disorder (478 pt-yr); 262 controls	Panic disorder as chronic stressor	Incidence
Vita et al. (2015) [21]	Italy	Observational cohort (21 y)	58 GD with stress-preceded onset	Stressful-event/impact assessment	Onset and recurrence
Yoshiuchi et al. (1998b) [22]	Japan	Prospective cohort	230 GD (182 F, 48 M)	Life events; daily hassles	Course (ATD)
Fukao et al. (2003) [23]	Japan	Prospective cohort	69 ATD-treated GD	Emotional stress and personality	Course

**Table 3:** Principal findings and direction of association for each included study. *Effect specific to Graves’ disease. CI, confidence interval; RR, relative risk; TNG, toxic nodular goiter; LES, Life Experiences Survey; TPO, thyroid peroxidase; F, female

Study (year) [ref]	Outcome	Key result	Direction
Winsa et al. (1991) [7]	Onset	Odds ratio 6.3 (95% CI 2.7-14.7) for the highest negative life-event score.	Positive
Sonino et al. (1993) [13]	Onset	Life events in the year before onset were more frequent than in controls.	Positive
Kung (1995) [14]	Onset	More patients than controls reported negative events (*P* < 0.0005).	Positive
Radosavljević et al. (1996) [15]	Onset	More life events in the year before diagnosis (*P* = 0.0001).	Positive
Matos-Santos et al. (2001) [16]	Onset	Negative events greater in GD than TNG or controls (*P* = 0.001); TNG ≈ controls.	Positive*
Topcu et al. (2012) [17]	Onset	Holmes-Rahe not significant; LES negative-event burden higher in GD.	Mixed
Lee et al. (2003) [18]	Onset	Life events linked to hyperthyroidism and to anxiety and depression.	Positive
Yoshiuchi et al. (1998) [19]	Onset	Life events raised GD risk in women (RR 7.7), not men.	Positive (F)
Chiovato et al. (1998) [20]	Incidence	No GD over 478 patient-years of chronic endogenous stress.	Null
Effraimidis et al. (2012) [8]	De novo	No link to de novo TPO antibodies or overt autoimmune disease.	Null
Vita et al. (2015) [21]	Onset and recurrence	Relapsers had a greater perceived stress impact than remitters.	Positive
Yoshiuchi et al. (1998b) [22]	Course	Daily hassles predicted persistent hyperthyroidism in women (RR 3.9).	Positive (F)
Fukao et al. (2003) [23]	Course	Chronic stress associated with a less favorable course, mainly in women.	Positive

Retrospective Case-Control Evidence on Disease Onset

The retrospective case-control literature was largely concordant in reporting an excess of antecedent stressful life events among patients developing GD. In the population-based study of Winsa et al., 208 patients with newly diagnosed GD reported significantly more negative life events in the 12 months before diagnosis than 372 matched controls, with an odds ratio of 6.3 (95% CI 2.7-14.7) for the highest category of negative life-event score; a family history of thyroid disease was independently associated with disease (odds ratio 3.6) [7]. Using the same semi-structured methodology in Italy, Sonino et al. found that life events in the year preceding onset were significantly more frequent in 70 patients with GD than in matched controls [13]. In a case-control study of 95 newly diagnosed Hong Kong patients, Kung reported that significantly more patients than controls had experienced negative life events (*P* < 0.0005), whereas positive and neutral events did not differ [14]. Radosavljević et al. corroborated this pattern in 100 newly diagnosed patients and 100 matched controls, documenting significantly more life events in the year before diagnosis (*P* = 0.0001) and elevated relative risks for specific stressors such as marked changes in workload and unemployment [15].

Two studies addressed the specificity of the association to autoimmune disease by including a non-autoimmune hyperthyroid comparator. Matos-Santos et al. compared 31 patients with GD, 31 with toxic nodular goiter and 31 healthy controls, and found that patients with GD had both a greater total number of stressful life events (174 versus 79 versus 50 events, respectively; *P* = 0.016) and a greater number and impact of negative events (141 versus 65 versus 27; *P* = 0.001), whereas the toxic nodular goiter group did not differ significantly from controls [16]. Topcu et al. similarly compared GD, toxic nodular goiter and healthy groups; although scores on the Holmes-Rahe scale did not differ significantly, patients with GD reported a significantly greater number and impact of negative events on the Life Experiences Survey, indicating an association most evident for the autoimmune phenotype and sensitive to the instrument used [17]. Lee et al., studying a Taiwanese population, reported that stressful life events were associated with hyperthyroidism and with accompanying anxiety and depressive symptomatology [18].

Sex-Specific Associations

Several studies indicated that the association between stress and GD onset is concentrated in women. Yoshiuchi et al. investigated 228 newly diagnosed Japanese patients (182 women and 46 men) with matched controls and, using multivariate analysis, found that stressful life events in the 12 months before diagnosis were significantly associated with the risk of GD in women, with a relative risk of 7.7 for the highest versus the lowest stress-score category, whereas no significant association was observed in men; daily-hassle scores did not differ between patients and controls [19]. Notably, cigarette smoking emerged as an independent risk factor in the same female analysis, indicating that stress operated alongside, rather than in place of, an established environmental trigger. This sex-restricted pattern was not confined to disease onset: in the analyses of clinical course described below, daily hassles predicted persistence of hyperthyroidism at 12 months in women but not in men [22], and a less favorable prognosis under antithyroid drug therapy was likewise observed predominantly among women [23]. The concentration of the association in women is congruent with the pronounced female preponderance of GD itself and with the sexual dimorphism of both HPA-axis reactivity and autoimmune susceptibility, although in none of these studies was the sex difference formally tested by a statistical test of interaction, so the apparent sex specificity should be regarded as hypothesis-generating rather than established.

Prospective and Endogenous Stress Evidence

The prospective evidence stands in contrast to the retrospective signal. Effraimidis et al. nested two case-control analyses within a prospective cohort of 790 euthyroid women who were first- or second-degree relatives of patients with autoimmune thyroid disease, assessing recent life events, daily hassles and affect at annual intervals; they detected no association between stress exposure and either the de novo appearance of thyroid peroxidase antibodies, one of the earliest events in thyroid autoimmunity, or the subsequent development of overt autoimmune thyroid disease [8]. A complementary line of evidence comes from Chiovato et al., who used chronic endogenous stress as a natural experiment: across 87 patients with panic disorder followed for a cumulative 478 patient-years, with a matched control group of 262 subjects, no case of GD or hyperthyroidism emerged, and the prevalence of thyroid autoantibodies was not increased [20]. Together, these studies indicate that when stress is measured prospectively and independently of the disease, the association observed in retrospective designs is not reproduced.

Stress and the Clinical Course of GD

Three studies examined whether stress influences the course of established disease. Vita et al., in an observational series accumulated over 21 years, studied 58 patients in whom the onset of GD had been preceded by at least one stressful event and found that those who subsequently experienced an exacerbation or relapse had a significantly greater perceived stress impact than those who remitted, implicating stress in both the initiation and the recurrence of hyperthyroidism [21]. In a prospective study of 230 newly diagnosed patients (182 women, 48 men), Yoshiuchi et al. reported that daily hassles measured six months into antithyroid drug therapy were independently associated with a persistent hyperthyroid state at 12 months in women (relative risk 3.9), but not in men [22]. Fukao et al. likewise found, in 69 antithyroid-drug-treated patients, that chronic psychological stress was associated with a less favorable disease course, again most clearly in women [23]. The course literature thus mirrors the onset literature both in its overall direction and in its female predominance, while sharing many of its methodological constraints.

Risk of Bias Within Studies

The results of the Newcastle-Ottawa appraisal are presented in Table 4. Total scores ranged from 5 to 9 of a possible nine stars. Applying the predefined thresholds, eight studies were rated at low risk of bias and five at moderate risk; none met the criteria for high risk. The recurring threat to validity was the retrospective ascertainment of stress in the case-control studies, in which patients were asked to recall life events after diagnosis; such designs are susceptible to recall bias, since heightened symptom-driven rumination may increase the reporting or salience of past adversity, and to reverse causation, because the hyperthyroid prodrome can itself generate stress. Studies that compared GD with non-autoimmune hyperthyroid controls [16,17] or that measured exposure prospectively and independently of the outcome [8,20] were judged least vulnerable to these biases and are correspondingly more informative for causal inference, notwithstanding that the prospective studies did not confirm an association. Comparability of cases and controls, and the validity of the exposure instruments, were generally adequate where reported.

**Table 4:** Newcastle-Ottawa Scale (NOS) appraisal of the included studies. Case-control studies were rated on selection, comparability, and exposure; cohort studies on selection, comparability, and outcome  [11]. Risk-of-bias categories were assigned using predefined thresholds: 7-9 stars, low risk; 4-6 stars, moderate risk; 0-3 stars, high risk.

Study (year) [ref]	Selection (max 4)	Comparability (max 2)	Exposure / outcome (max 3)	Total (max 9)	Risk of bias
Winsa et al. (1991) [7]	4	2	2	8	Low
Effraimidis et al. (2012) [8]	4	2	3	9	Low
Sonino et al. (1993) [13]	3	1	2	6	Moderate
Kung (1995) [14]	3	2	2	7	Low
Radosavljević et al. (1996) [15]	3	2	2	7	Low
Matos-Santos et al. (2001) [16]	3	2	2	7	Low
Topcu et al. (2012) [17]	3	1	2	6	Moderate
Lee et al. (2003) [18]	3	1	2	6	Moderate
Yoshiuchi et al. (1998) [19]	3	2	2	7	Low
Chiovato et al. (1998) [20]	3	1	3	7	Low
Vita et al. (2015) [21]	2	1	2	5	Moderate
Yoshiuchi et al. (1998) [22]	3	2	3	8	Low
Fukao et al. (2003) [23]	3	1	2	6	Moderate

Certainty of the Evidence

The GRADE assessment is summarized in Table 5. For both the onset and the course or recurrence of GD, the certainty of evidence was rated very low [12]. Beginning at low certainty by virtue of the observational designs, the onset evidence was rated down for serious risk of bias, predominantly retrospective exposure ascertainment with attendant recall bias and reverse causation; for serious inconsistency, reflecting the divergence between the positive retrospective studies and the null prospective findings; and for imprecision arising from several small studies with wide confidence intervals. Although some studies reported large effect estimates that might ordinarily warrant an upgrade, this was not applied in the presence of unresolved inconsistency and the likelihood that the effect magnitude is inflated by recall bias. The course and recurrence evidence, though directionally consistent, rested on only three small studies and was likewise downgraded for risk of bias and imprecision.

**Table 5:** GRADE certainty-of-evidence assessment by outcome. Source: [12]. Bodies of observational evidence began at low certainty and were rated down for the domains indicated. ⊕○○○ denotes very low certainty. GRADE, Grading of Recommendations Assessment, Development, and Evaluation

Outcome	Studies (design)	Risk of bias	Inconsistency	Indirectness	Imprecision	Certainty (GRADE)
Onset of Graves’ disease	11 (8 case-control, 3 cohort)	Serious	Serious	Not serious	Serious	Very low ⊕○○○
Course/recurrence	3 (cohort/observational)	Serious	Not serious	Not serious	Serious	Very low ⊕○○○

Discussion

This systematic review assembled 13 original observational studies addressing whether psychological and physiological stress acts as a trigger for GD. The central finding is a genuine tension in the evidence base. On one side stands a broadly consistent body of retrospective case-control research indicating that patients developing GD report an excess of negative stressful life events in the year before diagnosis, sometimes with strikingly large effect estimates [7,13-16,19]. On the other side stands a smaller but methodologically stronger prospective literature that fails to reproduce the association when stress is measured before, and independently of, the disease [8,20]. An honest synthesis must hold these two observations together rather than privileging the more numerous or the more intuitively appealing evidence.

Comparison With Previous Literature

The retrospective signal is notable for its consistency across diverse populations and instruments, from the Swedish population-based cohort of Winsa et al. [7] to replications in Italy [13], Hong Kong [14], Serbia [15], Japan [19], Portugal [16], and Taiwan [18]. Two features strengthen the internal credibility of this pattern. First, the association displayed biological specificity: where patients with GD were compared with patients with non-autoimmune toxic nodular goiter as well as with healthy individuals, the excess of stressful events was confined to the autoimmune group [16,17]. If stress were merely a non-specific correlate of thyrotoxicosis or of medical help-seeking, the toxic nodular goiter group would be expected to show a comparable excess, which it did not. Second, the association was repeatedly concentrated in women, both for onset [19] and for disease course [22,23].

The present findings are broadly consistent in direction with the 2023 systematic review and meta-analysis of Wang et al., which pooled 13 studies and reported a large summary effect favoring an association between stress and GD onset [9]. The two syntheses differ importantly in interpretation, however. Where that review combined heterogeneous exposure instruments and outcome metrics into a single standardized effect size, the present review judged such pooling to be methodologically unsafe and instead retained the distinction between designs, which is precisely where the evidence diverges. The consequence is a more conservative conclusion: the same primary literature, appraised with GRADE, supports only very low certainty, and the discrepancy between retrospective and prospective findings is treated here as a substantive signal about bias rather than as statistical heterogeneity to be absorbed into a random-effects model.

Ecological and natural-experiment observations have frequently been marshalled in support of a causal role. The most cited is the report by Paunkovic et al. of a steep rise in the incidence of GD in the Timok region of eastern Serbia during the civil war in the former Yugoslavia, where annual registrations climbed from an average of 16 new cases in the 1970s to 100 in 1995 and 148 in 1996, during a period of extreme collective stress [24]. Anecdotal support comes from a single published case report describing GD emerging and relapsing in close temporal relation to acute emotional stress [25]; as an uncontrolled observation in one patient, it carries no inferential weight beyond illustrating the clinical phenomenon. Such data are evocative and consistent with the retrospective case-control findings, but they are ecological or anecdotal in nature and cannot exclude the many co-varying wartime exposures, including iodine supply, infection, nutrition, smoking and altered health-care access, that also influence thyroid autoimmunity [3].

The mechanistic scaffolding for a causal interpretation is well developed. Stressors activate the HPA axis and the sympathoadrenomedullary system, and the resulting glucocorticoid and catecholamine surges reshape immune function in ways that are dose- and time-dependent [4]. Chronic activation, through the accumulation of allostatic load, can tip regulatory set-points and disturb the equilibrium of adaptive immunity [5]. In thyroid autoimmunity specifically, stress hormones have been proposed to bias the Th1/Th2 balance toward the Th2-dominated, antibody-driven response characteristic of GD and to interfere with regulatory T-cell control [6]. Preclinical evidence supports these pathways: in a murine model of chronic stress, sustained stress exposure lowered circulating triiodothyronine and attenuated humoral alloimmune responses, demonstrating that stress can modulate immunity through the thyroid axis, although extrapolation from rodent models to human autoimmune disease is necessarily indirect [26]. Reviews devoted to the emotional-stress hypothesis have reached similar conclusions while emphasizing that mechanistic plausibility is not itself evidence of causation and that intervention data remain conspicuously absent [2].

Interpreting the Discrepancy Between Designs

The prospective evidence is where the causal narrative encounters its most serious challenge. Effraimidis et al. followed 790 euthyroid women at genetic risk and measured stress prospectively and repeatedly, finding no relationship with the de novo appearance of thyroid peroxidase antibodies or with progression to overt disease [8]. Because autoantibody seroconversion precedes clinical hyperthyroidism, often by years, this design largely circumvents the reverse-causation problem that bedevils retrospective work. The convergent finding of Chiovato et al., that chronic endogenous stress in panic disorder sustained across hundreds of patient-years did not precipitate GD or raise autoantibody prevalence, uses an entirely different exposure model to reach the same null conclusion [20].

The most parsimonious reconciliation is that much of the retrospective association reflects recall bias and reverse causation. The insidious neuropsychiatric prodrome of hyperthyroidism, comprising irritability, insomnia and emotional lability, may both generate genuine interpersonal stress and heighten the retrospective salience of ordinary life events, inflating the apparent exposure in cases relative to controls. This interpretation does not require that stress be causally inert, but it cautions against reading the case-control effect sizes at face value. Measurement heterogeneity compounds the problem. Topcu et al. found no difference between patients and controls on the Holmes-Rahe scale yet a significant excess of negative events on the Life Experiences Survey within the very same sample [17], demonstrating that the direction of a study’s conclusion can hinge on the instrument selected. A further difficulty is confounding by co-occurring environmental triggers: stress rarely occurs in isolation from smoking, altered iodine intake, infection and, in women, pregnancy and the postpartum state, each of which independently modifies risk [1,3].

Clinical Implications

For the clinician, it is defensible to acknowledge that stressful life circumstances may plausibly contribute to the emergence and course of GD in the setting of genetic susceptibility, while being careful neither to imply that patients have caused their own illness nor to promise therapeutic benefit from stress reduction that the evidence does not yet support. The evidence on disease course is of particular clinical interest, since if stress modulates the tempo of established disease [21-23], stress reduction becomes a plausible adjunct to conventional therapy. It must be stated candidly, however, that no adequately powered randomized trial has demonstrated that a stress-management intervention improves remission rates or antibody titers in GD, and current mechanistic reviews identify this as the critical evidential gap [2]. Recommendations to manage stress in GD are therefore reasonable and low-risk, but they rest on association rather than on demonstrated efficacy.

Future Research Directions

The priorities for future research follow directly from the limitations of the existing evidence. The field requires adequately powered prospective cohort studies that measure stress before disease onset using standardized, validated instruments applied consistently across cohorts, so that findings are not contingent on the choice of scale. Such studies should incorporate objective biological indices of stress-system activation, including diurnal salivary cortisol, hair cortisol as an integrated marker of chronic exposure, and autonomic measures such as heart rate variability, alongside serial thyroid autoantibody assessment, so that exposure is ascertained independently of the outcome and is not vulnerable to recall. Recruitment of at-risk cohorts, such as antibody-negative first-degree relatives, would permit capture of the earliest transition to autoimmunity. Analyses should be prespecified with formal tests of interaction by sex and should adjust for smoking, iodine status, infection, and reproductive events. Mendelian randomization and other quasi-experimental approaches may help triangulate causation where randomization is impossible, and randomized trials of structured stress-reduction interventions in established GD would directly test whether the association is therapeutically actionable.

Strengths and Limitations

The strengths of this review include a broad, multi-database search conducted according to the PRISMA 2020 statement, explicit PICOS-based eligibility criteria, duplicate study selection and data extraction, formal risk-of-bias appraisal with predefined thresholds, GRADE certainty rating, and a synthesis that gives the inconsistent prospective evidence its due weight rather than emphasizing only the positive case-control findings.

Several limitations temper the conclusions. First, and most importantly, the constituent evidence is dominated by retrospective case-control studies whose ascertainment of stress after diagnosis renders them vulnerable to recall bias and reverse causation. Second, the included studies were clinically and methodologically heterogeneous, differing in stress instruments, exposure windows, comparators, and outcome metrics, which precluded a valid quantitative meta-analysis and obliged a narrative synthesis; the direction of effect could be compared across studies, but a pooled magnitude could not be responsibly estimated. Third, most studies were modest in size, and several did not report adjusted effect estimates, limiting control of confounders such as smoking, iodine status, and family history. Several also did not report exact comparison-group sizes, so the cumulative participant total is a conservative minimum. Fourth, publication and reporting biases may have favored positive findings, and because formal small-study diagnostics were not applicable, this possibility could be addressed only qualitatively. Fifth, the review protocol was not prospectively registered with PROSPERO, which reduces methodological transparency, although the protocol was finalized before the search commenced and the review followed established systematic review methodology. Finally, inter-reviewer agreement was not quantified using a chance-corrected statistic.

## Conclusions

The available observational evidence indicates that antecedent stressful life events are associated with the onset, and to a lesser extent, the clinical course of GD in genetically predisposed individuals, a biologically plausible association repeatedly demonstrated across populations, apparently specific to autoimmune hyperthyroidism, and concentrated in women. This association derives largely from retrospective studies that are inherently susceptible to recall bias and reverse causation, and it is not corroborated by the limited prospective evidence, in which stress measured independently of the disease showed no relationship to incident thyroid autoimmunity; the overall certainty of evidence is accordingly very low. Stress is therefore best regarded, on current evidence, as a plausible but unproven contributory trigger rather than an established cause, and this review makes no causal claim. Resolving the question is now primarily a matter of study design rather than of accumulating further retrospective series. The priority is adequately powered prospective cohort studies that enroll at-risk, antibody-negative individuals, measure stress before disease onset using standardized and consistently applied instruments together with objective biomarkers such as diurnal and hair cortisol, follow participants with serial autoantibody testing, and prespecify tests of interaction by sex. These should be complemented by randomized trials of structured stress-reduction interventions in established GD to determine whether the relationship is not only causal but also therapeutically actionable.

## Appendices

Appendix 

**Table 6:** Complete database-specific search strategies and record counts

Database	Search string	Records
PubMed/MEDLINE	(("Stress, Psychological"[Mesh]) OR ("Life Change Events"[Mesh]) OR (stressful life event[tiab]) OR (psychological stress[tiab]) OR (emotional stress[tiab])) AND (("Graves Disease"[Mesh]) OR (Graves disease[tiab]) OR (toxic diffuse goit[tiab]) OR (autoimmune hyperthyroidism[tiab]) OR (thyrotoxicosis[tiab]))	68
Embase	('mental stress'/exp OR 'life event'/exp OR 'stressful life event':ti,ab OR 'psychological stress':ti,ab OR 'emotional stress':ti,ab) AND ('Graves disease'/exp OR 'Graves disease':ti,ab OR 'toxic diffuse goit':ti,ab OR 'autoimmune hyperthyroidism':ti,ab OR 'thyrotoxicosis':ti,ab)	73
Scopus	TITLE-ABS-KEY(("stressful life event" OR "psychological stress" OR "emotional stress" OR "life change event") AND ("Graves disease" OR "toxic diffuse goit*" OR "autoimmune hyperthyroidism" OR thyrotoxicosis))	42
Web of Science	TS=(("stressful life event" OR "psychological stress" OR "emotional stress" OR "life change event") AND ("Graves disease" OR "toxic diffuse goit*" OR "autoimmune hyperthyroidism" OR thyrotoxicosis))	34
PsycINFO	(DE "Stress" OR DE "Life Experiences" OR "stressful life event*" OR "psychological stress") AND ("Graves disease" OR "autoimmune hyperthyroidism" OR thyrotoxicosis)	48
Cochrane Library	("stressful life event*" OR "psychological stress" OR "emotional stress") AND ("Graves disease" OR "autoimmune hyperthyroidism" OR thyrotoxicosis)	14
Total		279
